# The Beneficial Effect of IL-12 and IL-18 Transduced Dendritic Cells Stimulated with Tumor Antigens on Generation of an Antitumor Response in a Mouse Colon Carcinoma Model

**DOI:** 10.1155/2022/7508928

**Published:** 2022-03-25

**Authors:** Jagoda Mierzejewska, Katarzyna Węgierek-Ciura, Joanna Rossowska, Agnieszka Szczygieł, Natalia Anger-Góra, Bożena Szermer-Olearnik, Magdalena Geneja, Elżbieta Pajtasz-Piasecka

**Affiliations:** Hirszfeld Institute of Immunology and Experimental Therapy, Polish Academy of Sciences, Wroclaw, Poland

## Abstract

The main purpose of our study was to determine the effect of dendritic cell (DC) transduction with lentiviral vectors carrying sequences of *il18* and/or *il12* genes on the level of antitumor activity *in vitro* and *in vivo*. We examined the ability of DCs to migrate to the tumor-draining lymph nodes and infiltrate tumor tissue and to activate the local and systemic antitumor response. On the 15th day, DCs genetically modified for production of IL-12 and/or IL-18 were administered peritumorally to C57BL/6 female mice with established MC38 tumors. Lymphoid organs and tumor tissue were collected from mice on the 3rd, 5th, and 7th days after a single administration of DCs for further analysis. Administration of DCs transduced for production of IL-12 alone and in combination with IL-18 increased the inflow and activity of CD4^+^ and CD8^+^ T lymphocytes in the tumor microenvironment and tumor-draining lymph nodes. We also found that even a single administration of such modified DCs could trigger a systemic antitumor response as well as inhibit tumor growth. Application of the developed DC-based vaccines may exert a favorable impact on stimulation of an antitumor immune response, especially if these DC vaccines are administered repeatedly.

## 1. Introduction

The tumor microenvironment (TME) is a complex network of interactions between cellular and non-cellular components, enabling cancer progression. These interactions are dynamic “two-way” processes, including both direct cell-to-cell contact and the effect of soluble factors in the TME [1, 2].

Dendritic cell- (DC-) based vaccines are an example of immunotherapy that causes TME changes [3, 4]. In the tumor environment, DCs play a special role in activating antigen-specific cytotoxic T lymphocytes (CTLs) by recognizing antigens released by the tumor and presenting them in situ during the cross-presentation process [5]. However, the process of T cell activation in the TME is strictly dependent on the DC differentiation status as well as the place of DC infiltration [6]. *Ex vivo* stimulation of DCs influences the initiation of the maturation process of these cells and thus the morphological, phenotypic, and functional changes of these cells. Maturing DCs migrate to lymph nodes, where they present antigens to naïve T cells [7–11]. The use of dendritic cells in immunotherapy assumes the induction of the response of the immune system as a result of the effective presentation of tumor antigens. Although dendritic cell vaccines have recently been introduced into clinics and are considered nontoxic and relatively safe, there are still many questions to be cleared. The research is aimed at shortening the time-consuming *ex vivo* DC propagation procedure and at finding the best form of antigen loading of dendritic cells. DC vaccines are considered nontoxic and relatively safe.

Strong stimulation of DCs leads to the secretion of Interleukin-12—a cytokine also produced by monocytes, macrophages, and B lymphocytes. IL-12 is a heterodimer composed of a light chain, also referred to as p35 or IL-12*α*, and a heavy chain—p40 or IL-12*β* [12]. This cytokine is involved in induction of both the cellular and the humoral immune response. IL-12 stimulates the proliferation and activates the functions of NK cells, CTLs, and Th1 cells and thus the secretion of IFN-*γ* by these cells. On the other hand, it is responsible for the proliferation of B cell precursors and their differentiation into IgG2a-producing cells. It also plays an important role in the inhibition of angiogenesis. Additionally, IL-12 affects tumor cells, causing increased expression of the antigen MHC I on their surface, which may facilitate tumor identification and promote the cytotoxic activity of lymphocytes [13].

Interleukin 18 is a cytokine functionally similar to IL-12. It is produced by monocytes, macrophages, DCs, T cells, and B cells as well as keratinocytes, epithelial cells, and osteoblasts [14]. IL-18 was initially identified as a cytokine that facilitated the production of IFN-*γ* [15]. This cytokine even more strongly than IL-12 stimulates production of IFN-*γ* by T cells and NK cells. Furthermore, IL-18 induces secretion of IL-3, IL-9, and IL-13 by CD4^+^ T cells, NK cells, basophils, and mast cells. It also enhances the cytotoxic activity of CD8^+^ T lymphocytes, CD4^+^ T cells, and NK cells. The effect of IL-18 on immune response modulation depends, to a large extent, on the presence of IL-12 in the environment. The synergistic action of these two cytokines leads to the induction of a Th1-type immune response. In contrast, during the absence of IL-12 in the environment, IL-18 promotes a Th2-type immune response [16]. Hence, administration of IL-18 is associated with septic shock-like severe toxicity that prevents its application [14]. What is more, the first clinical studies demonstrated that systemic administration of IL-12 is associated with serious side effects (e.g., fever, chills, decreased peripheral blood cells, and organ dysfunction) [13].

The side effects of these immunotherapeutic agents can be reduced by using alternative methods of administration, e.g., by gene therapy. The gene therapy method involves delivering the genetic material encoding, e.g., cytokine genes directly to the tumor tissue using plasmids, mRNA, viruses, or transduced cells capable of expressing cytokines when administered locally. One of the most frequently used and effective methods for gene insertion into cells or tissues is lentiviral vector-based transduction. The vectors can effectively infect both dividing and non-dividing cells, which makes them particularly attractive for use in gene therapy [17].

Thus, the use of dendritic cells stimulated with tumor antigens for transduction with cytokine genes may be a promising tool in immunotherapy. Numerous approaches using vaccination with IL-12 single-transduced DCs [18, 19] and IL-18 single-transduced DCs [20–23] in cancer immunotherapy have been reported. The current pre-clinical and clinical studies with the use of non-neoplastic cells transduced for the production of IL-12, such as dendritic cells, fibroblasts, macrophages, and mesenchymal stem cells (MSCs) used in therapy as adjuvants, prompted us to further work on this issue and use the synergistic effect of IL-12 and IL-18 [24].

The main purpose of our study was to determine the effect of DC transduction with lentiviral vectors carrying the sequences of *il18* and/or *il12* genes on the level of antitumor activity of these cells both *in vitro* and *in vivo*. One of the most important goals of our study was to examine the ability of DCs peritumorally inoculated into MC38 colon carcinoma-bearing mice to migrate to the tumor-draining lymph nodes and infiltrate tumor tissue and to activate the local and systemic antitumor response. DCs simultaneously producing IL-18 and IL-12 (DC/IL-18 + IL-12/TAg) were characterized by the highest maturity and degree of activity. Moreover, they infiltrated the tumor tissue most quickly and most efficiently. DCs producing IL-18 and/or IL-12 also induced activation of CD4^+^ and CD8^+^ T cells in lymph nodes and high infiltration of CD4^+^ and CD8^+^ T cells to the tumor tissue. Splenocytes obtained from DC/IL-18 + IL-12/TAg treated mice showed more efficient cytotoxic activity towards MC38 tumor cells compared to spleen cells from MC38 control mice. The use of the developed DC/IL-18 + IL-12/TAg vaccine cells in immunotherapy allowed for a statistically significant inhibition of MC38 cancer tumor growth.

The obtained results confirm that DCs transduced for the simultaneous production of IL-18 and IL-12, used as cell vaccines, contribute to the generation of an efficient antitumor immune response.

## 2. Materials and Methods

### 2.1. Preparation of DC Vaccines

**Figure 1:**
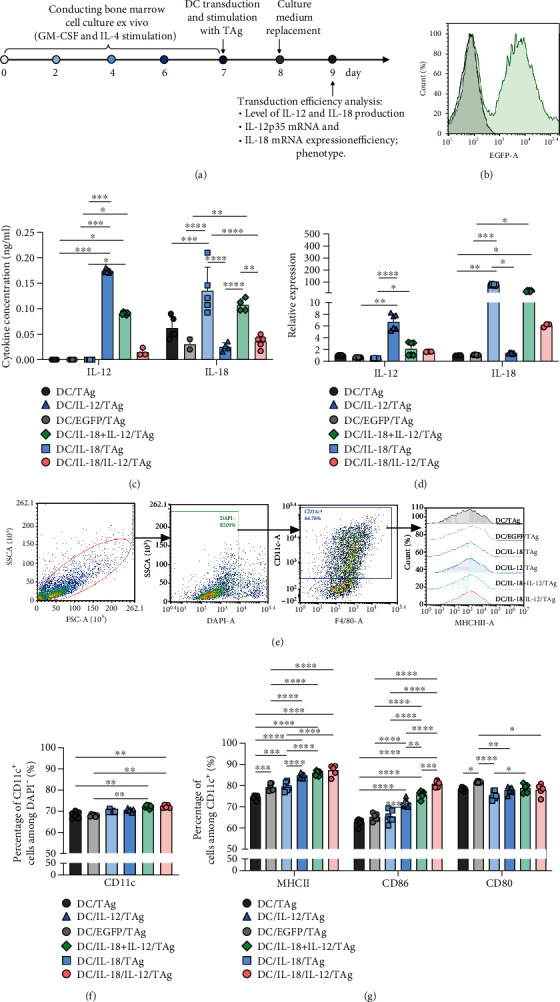
The influence of lentiviral transduction with *il12* and *il18* genes on bone marrow-derived dendritic cells (DCs). (a) Preparation schedule of genetically modified DCs. (b) Expression of EGFP by DC/TAg cells. Control DC/TAg cells (black histogram) and DC/EGFP/TAg (green histogram). (c) Concentration of IL-12 and IL-18 in supernatants collected on the 9th day of DC culture measured using ELISA. (d) Relative expression of murine IL-12p35 mRNA and murine IL-18 mRNA in transduced cells on the 9th day of DC culture measured by real-time PCR. (e) Scheme of phenotype analysis of vaccine cells. (f) Percentage of CD11c^+^ cells on the 8th day of DC cultured. (g) Expression of MHC II and costimulatory molecules (CD86 and CD80) on the surface of CD11c^+^ cells, on the 9th day of culture, measured using flow cytometry. Results are presented as mean + SD calculated for at least three repeats in two independent experiments. Differences between groups were estimated using the nonparametric Kruskal-Wallis test followed by Dunn's multiple comparison post hoc test ((c) concentration of IL-12; (d, f)) or one-way ANOVA followed by Tukey's multiple comparison post hoc test ((c) concentration of IL-18; (g)) (^∗^*p* < 0.05, ^∗∗^*p* < 0.01, ^∗∗∗^*p* < 0.001, ^∗∗∗∗^*p* < 0.0001).

DCs were differentiated *ex vivo* from the bone marrow of healthy C57BL/6 mice according to the procedure described by Rossowska et al. [25]. DCs were cultured in 75 cm^2^ nontreated flasks (Eppendorf) in RPMI 1640 (Gibco) supplemented with 100 U/ml penicillin (Sigma-Aldrich), 100 mg/ml streptomycin (Sigma-Aldrich), 0.5% sodium pyruvate (Sigma-Aldrich), 2-mercaptoethanol (Sigma-Aldrich), here named as the culture medium (CM), supplemented with 10% of fetal bovine serum (FBS, Sigma-Aldrich), rmGM-CSF (40 ng/ml, ImmunoTools), and rm IL-4 (10 ng/ml, ImmunoTools). On the 6th day, loosely attached immature DCs were collected and put in new flasks. On the next day, cells were transduced with lentiviral vectors and after 4 hours stimulated with tumor antigens (TAg). Based on the collected data, while optimizing the transduction method, we decided to use lentiviral vectors in the amount of 4 virus infectious particles per 1 dendritic cell. The thawed tumor lysate was added to the dendritic cell culture in an amount of 10% of the volume of the culture medium. The cells were incubated for 24 h with virus-containing supernatants and 8 *μ*g/ml polybrene (Sigma). On the 8th day, DCs were collected and used in further *in vitro* experiments or as DC-based vaccines. The preparation schedule of DC vaccines is shown in Figure 1(a).

### 2.2. Vector Production

The Lenti-X 293T cell line (Clontech) was maintained in high-glucose Dulbecco's Modified Eagle Medium (Gibco) supplemented with 100 U/ml penicillin (Sigma-Aldrich), 100 mg/ml streptomycin (Sigma-Aldrich), 0.5% sodium pyruvate (Sigma-Aldrich), and 10% FBS (Sigma-Aldrich). Lenti X cells were cultured in 75 cm^2^ flasks (Corning) and passed every 2–3 days. Lentiviral vectors were produced using a 3rd generation lentiviral system consisting of pMDLg/pRRE, pRSV-Rev, pMD2.G (the plasmids were a gift from Didier Trono (Addgene plasmid # 12251, 12253, 12259)), and pLV/mIL-12, plasmid encoding murine *il12* gene (p35 and p40 subunits); pLV/mIL-18, plasmid encoding murine *il18* gene; pLV/mIL-18/mIL-12, plasmid encoding murine il-18 and *il12* gene (p35 and p40 subunits) simultaneously; and pLV/EGFP, control plasmid encoding enhanced green fluorescent protein (EGFP). Lentiviral vectors used for cell modifications were produced as previously described by Rossowska et al. [26]. LV-containing supernatant was collected and concentrated by PEG 6000 (Sigma-Aldrich) precipitation. The pellet containing lentiviral vectors was suspended in PBS and stored at -80°C. The titer of the lentiviral vectors was evaluated using the commercially available QuickTiter Lentivirus Titer Kit according to the manufacturer's instructions (Cell Biolabs).

### 2.3. Tumor Cell Culture

The *in vivo* growing cell line of MC38 murine colon carcinoma from the Tumor Bank of the TNO Radiobiology Institute, Rijswijk, Holland, was adapted to *in vitro* conditions as described by Pajtasz-Piasecka et al. [27]. MC38 was maintained in culture medium supplemented with 5% of FBS (Sigma-Aldrich). MC38 cells were cultured in 75 cm^2^ flasks (Corning) and passed every 2–3 days. MC38 cell lysate (named here tumor antigens (TAg)) was prepared from a MC38 cell suspension with a density of 5 × 10^6^/ml, which was frozen in liquid nitrogen and thawed at 37°C five times. Then, the mixture was sonicated for 2 h. The lysate was frozen and stored at -20°C.

### 2.4. Determination of IL-12 and IL-18 Expression Efficiency

The expression of *il12* and *il18* in genetically modified DC was measured 24 h after cell transduction by real-time PCR. Total RNA was isolated using a NucleoSpin RNA kit (Macherey-Nagel) and reverse-transcribed with a First Strand cDNA Synthesis Kit (Thermo Fisher). Real-time PCR was performed using TaqMan Universal PCR Master Mix and TaqMan Gene Expression Assay primers for *il12* and *il18* (Applied Biosystem) in reference to the hprt gene.

### 2.5. Determination of EGFP Expression Efficiency

The expression of EGFP in genetically modified DC was measured 24 h after cell transduction by the BD FACSDiva software version 8.0 and the NovoExpress software 1.3.0 (ACEA Biosciences, Inc.).

### 2.6. Analysis of Cytokine Production

The concentration of IL-12 and IL-18 was determined in supernatants from above 24-hour DC-transduced culture set on 12-well plates at a density of 0.5 × 10^6^ cells/1 ml/well in culture medium supplemented with 10% FBS and 40 ng/ml rm GM-CSF. Production of cytokines by DCs and spleen cells was evaluated using commercially available ELISA kits (IL-4, IL-10—BD Biosciences; IL-12, IL-18, IFN-*γ*—Invitrogen) according to the manufacturer's instructions.

### 2.7. Phenotype Characteristics of DC Vaccines

The flow cytometry method was applied to determine the percentage and mean fluorescence intensity of CD11c, MHC II, CD80, and CD86 on the surface of living DCs. Cells were labeled with monoclonal antibodies conjugated with fluorochromes: anti-CD11c BV650, anti-MHC II APC-Cy7, anti-CD80 PerCP-Cy5.5, and anti-CD86 PE-Cy7 all from BioLegend. The cells were stained for 45 min at 4°C. To identify dead cells, 50 *μ*l of DAPI dye solution (1 *μ*g/ml, Molecular Probes) was added to the samples immediately before analysis. The expression of cell surface markers was analyzed using a BD LSRFortessa Cell Analyzer (Becton Dickinson, Cat. No. 649225B5) with the BD FACSDiva software version 8.0 and the NovoExpress software 1.3.0 (ACEA Biosciences, Inc.).

### 2.8. Coculture of Splenocytes and Modified DCs *In Vitro*

Genetically modified DCs stimulated with tumor antigens were seeded in a 12-well plate (Nunc) at a density of 1.8 × 10^5^ cell/0.5 ml/well. Splenocytes obtained from C57BL/6 healthy female mice and stored in liquid nitrogen were thawed, centrifuged (7 min, 192 × g), resuspended in RPMI (Gibco) supplemented with 10% FBS(Sigma-Aldrich), and applied at a density of 1.8 × 10^6^ cells/0.5 ml/well to 12-well plates with DCs. The wells were supplemented with rh IL-2 (200 U/ml, ImmunoTools). Mixed culture of transduced DC and splenocytes was carried out for 5 days under standard conditions (37°C, 5% CO_2_). After that, the spleen cell phenotype and their cytotoxic activity as well as the concentration of cytokines in the supernatants collected from the 5-day mixed culture were evaluated.

### 2.9. Coculture of Splenocytes and MC38 Tumor Cells: Restimulation of Spleen Cells

Splenocytes obtained from mice were cocultured with mitomycin C-treated MC38 cells (50 mg mitomycin C/3 × 10^6^ cells, 30 min., 37°C, Sigma-Aldrich) in the presence of recombinant human IL-12 (200 U/ml, ImmunoTools). After 5 days of restimulation under standard conditions (37°C, 5% CO_2_), the spleen cells' phenotype and their cytotoxic activity as well as the concentration of cytokines in the supernatants collected from the 5-day mixed culture were evaluated.

### 2.10. CD107a Degranulation Assay

A suspension of stimulated splenocytes (2 × 10^5^ cells/well) was applied to flat-bottom 96-well plates (Nunc) with previously prepared single-layer MC38 cell culture (the ratio of tumor cells to splenocytes was 1 : 5). APC conjugated anti-CD107a monoclonal antibody, PMA (50 mg/ml, Sigma-Aldrich), ionomycin (1 *μ*g/ml, Sigma-Aldrich), and rh IL-2 (200 U/ml, ImmunoTools) were added to the wells. After 2 h at 37°C, cells were harvested and labeled with fluorochrome-conjugated monoclonal antibodies: anti-CD45 BV605, anti-CD4 FITC, anti-CD8 APC-Fire, and anti-NK1.1 PE-Dazzle (all from BioLegend). Cells were incubated with antibodies for 45 min at 4°C. To identify dead cells, 50 *μ*l of DAPI dye solution (1 *μ*g/ml, Molecular Probes) was added to the samples immediately before analysis. Samples were analyzed by a BD LSRFortessa Cell Analyzer (Becton Dickinson, Cat. No. 649225B5) with the BD FACSDiva software 8.0 and the NovoExpress software version 1.3.0 (ACEA Biosciences, Inc.).

### 2.11. Estimation of Spleen Cell Cytotoxicity

Target MC38 cells were labeled with DiOC18(3) (20 min, 37°C, Invitrogen) and applied to round-bottom 96-well plates (Greiner) at a density of 1 × 10^4^ cells/well. Five-day stimulated splenocytes were plated on target cells at a density of 1 × 10^5^ cells/well (ratio of effector cells to target cells was 10 : 1) or a density of 3 × 10^5^ cells/well (ratio of effector cells to target cells was 30 : 1). The rh IL-2 was added to each well (200 U/ml, ImmunoTools). After 4 hours, dead cells were stained with 7.5 nM propidium iodide solution (10 min, 37°C, Sigma-Aldrich). Samples were analyzed by a BD LSRFortessa Cell Analyzer (Becton Dickinson, Cat. No. 649225B5) with the BD FACSDiva software 8.0 and the NovoExpress software 1.3.0 (ACEA Biosciences, Inc.). The effector cell cytotoxic activity was determined as the percentage of dead target cells minus the percentage of dead control cells.

### 2.12. Mouse Treatment Schedule

**Figure 2:**
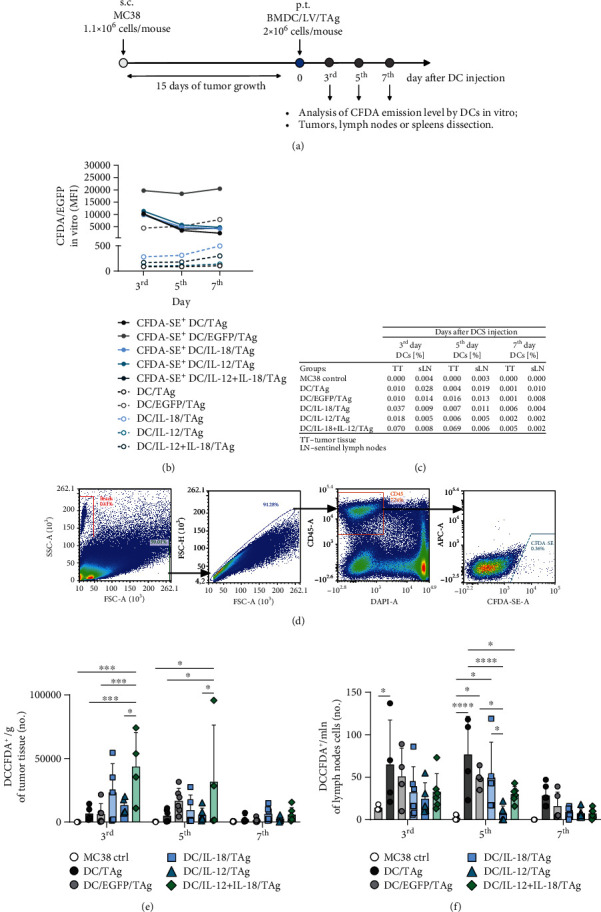
Analysis of the ability of CFDA-labeled DCs to migrate to the tumor-draining lymph nodes or infiltrate the tumor nodule of MC38 murine colon carcinoma. (a) Treatment schedule of an in vivo experiment. Mice with established tumors were administered peritumorally (p.t.) with genetically modified DCs, stimulated with TAg, and then fluorescently labeled with CFDA-SE. (b) The persistence of DC labeling with CFDA-SE was verified in in vitro conditions on the 3rd, 5th, and 7th days using flow cytometry. tLNs and tumor nodules were collected on the 3rd, 5th, and 7th days after administration of DCs. (c) Percentages of DCs in tumor nodules and tumor-draining lymph nodes. (d) Scheme of the cytometric analysis of CFDA-SE^+^ DCs in collected tissues (here representative data from tumor analysis). After elimination of debris, cell clumps, and dead cells, CD45^+^ cells were gated, and then among them, CFDA-SE labeled cells were identified. (e) Mean number + SD of DCs per 1 g of tumor weight. (f) Mean number + SD of DCs per 1 million cells isolated from tumor-draining lymph nodes. The total number of CFDA-SE^+^ DCs in tumor and tLN suspension was measured using counting beads. Results are presented as mean ± SD calculated for 4-6 mice per group. Differences between groups were estimated using the parametric two-way ANOVA followed by Tukey's multiple comparison post hoc test (e, f) (^∗^*p* < 0.05, ^∗∗^*p* < 0.01, ^∗∗∗^*p* < 0.001, ^∗∗∗∗^*p* < 0.0001).

The C57BL/6 female mice (Center of Experimental Medicine of the Medical University of Białystok, Poland) were subcutaneously (s.c.) inoculated in the right flank with MC38 colon carcinoma cells (1.1 × 10^6^ cells/mouse, day 0). The mice were treated according to the scheme presented in Figure 2(a). On the 15th day, mice with established tumors were administered peritumorally (p.t.) with DCs genetically modified for IL-12 and IL-18 coproduction (DC/IL-18/TAg, DC/IL-12/TAg, DC/IL-18 + IL-12/TAg), stimulated with MC38 tumor cell lysate (2 × 10^6^ cells/mice) or control cells (DC/TAg, DC/EGFP/TAg). To identify transduced cells in lymph nodes and tumor tissue, staining with a fluorescent dye CFDA-SE (Vybrant CFDA SE Cell Tracer Kit, Invitrogen) was performed. Lymphoid organs and tumor tissue were collected and homogenized from mice on the 3rd, 5th, and 7th days after a single administration of cell vaccines for further analysis. After the experiments, mice were sacrificed by cervical dislocation. All experiments were performed in accordance with EU Directive 2010/63/EU for animal experiments and were approved by the 1st Local Ethics Committee for Experiments with the Use of Laboratory Animals, Wroclaw, Poland (authorization no. 21/2017).

### 2.13. Staining and Evaluation of CFDA Fluorescence Intensity in Vaccine Cells *In Vitro*

Vaccine cells with a density of 5 × 10^6^ cells/ml were stained with CFDA-SE (Vybrant CFDA SE Cell Tracer Kit, Invitrogen) at a final concentration of 5 *μ*M. The staining procedure was carried out according to the manufacturer's instructions. The fluorescence intensity of CFSE stained DCs was determined using a BD LSRFortessa (Becton Dickinson) with the BD FACSDiva software.

### 2.14. Assessment of the Migration Capacity of Vaccine Cells

Labeling of DCs with CFDA-SE dye made it possible to determine the migration capacity of these cells from the injection site to the lymph nodes. Lymph node cells were stained with anti-CD45 V500 antibody (clone: 30-F11, BD Horizon, 45 min, 4°C, BioLegend). After 45 min of incubation, DAPI solution (1 *μ*g/ml) was added. Samples were analyzed by a BD LSRFortessa Cell Analyzer (Becton Dickinson, Cat. No. 649225B5) with the BD FACSDiva software 8.0 and the NovoExpress software 1.3.0 (ACEA Biosciences, Inc.)

### 2.15. Analysis of Myeloid Cells and Lymphocytes in Spleen, Tumor, and Lymph Nodes after Therapy

Splenocytes, lymph node cells, and tumor cells isolated from mice were incubated with anti-mouse CD16/CD32 mAb (15 min, 4°C, eBioscience). Splenocytes were stained with the LIVE/DEAD FixableViolet Dead Staining Kit (Thermo Fisher Scientific, Inc.). Subsequently, the cells were divided and stained with cocktails of fluorochrome-conjugated monoclonal antibodies: anti-CD3 PE-CF594, CD19 PE-CF594, and CD49b PE-CF594 (all from BD Biosciences); CD45 V500, CD11b PerCP-Cy5.5, CD11c BV650, F4/80 Alexa Fluor 700, CD86 PE-Cy7, and MHC II APC-Cy7 (all from BioLegend) for myeloid cell identification; and CD45 V500, CD4 PerCp-Cy5.5, CD8 PE-Cy7, CD49b PE-CF594, CD44 PE, and CD62L BV605 for lymphocyte identification. Cells were incubated with antibodies for 45 min at 4°C. Additionally, the percentage of Treg cells was determined among the splenocytes. For this purpose, the cells were fixed using the Foxp3/Transcription Factor Staining Buffer Set (eBioscience) and then incubated with anti-FoxP3 APC antibodies (eBioscience) [28]. To identify dead cells in lymph node cells and tumor cells, 50 *μ*l of DAPI dye solution (1 *μ*g/ml, Molecular Probes) was added to the samples immediately before analysis. The analysis was performed using a BD LSRFortessa Cell Analyzer (Becton Dickinson, Cat. No. 649225B5) with the BD FACSDiva software 8.0 and the NovoExpress software 1.3.0 (ACEA Biosciences, Inc.).

### 2.16. Therapeutic Treatment Schedule

**Figure 3:**
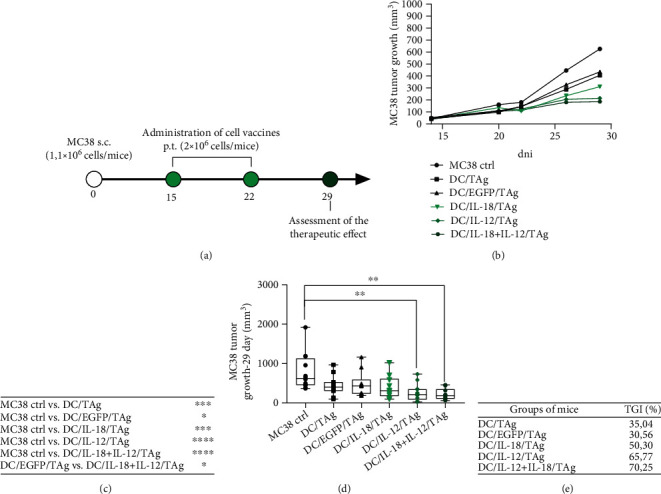
Impact of DC-based vaccines therapy on tumor growth. (a) The C57BL/6 female mice were subcutaneously (s.c.) inoculated in the right flank with MC38 colon carcinoma cells (1.1 × 10^6^ cells/mouse, day 0). On the 15th and 22nd days, mice with established tumors were administered peritumorally (p.t.) with DCs genetically modified for IL-12 and IL-18 coproduction (DC/IL-18/TAg, DC/IL-12/TAg, DC/IL-18 + IL-12/TAg), stimulated with MC38 tumor cell lysate (2 × 10^6^ cells/mice) or control cells (DC/TAg, DC/EGFP/TAg). On the 29th day, the therapeutic effect of the administration of vaccines was determined. (b) Graph presenting median tumor volume after immunotherapy. (c) Table presenting statistically significant differences between groups after immunotherapy. (d) Box graph presenting median tumor volume, calculated on the 29th day of the experiment. (e) Table presenting MC38 tumor growth inhibition (TGI) calculated on 29th day of experiment in relation to the MC38 ctrl group. Results are presented as mean ± SD calculated for 10-13 mice per group. Differences between groups were estimated using the nonparametric Kruskal-Wallis test followed by Dunn's multiple comparison post hoc test.

The C57BL/6 female mice were subcutaneously (s.c.) inoculated in the right flank with MC38 colon carcinoma cells (1.1 × 10^6^ cells/mouse, day 0). The mice were treated according to the scheme presented in Figure 3(a). On the 15th and 22nd day, mice with established tumors were administered peritumorally (p.t.) with DCs genetically modified for IL-12 and IL-18 coproduction (DC/IL-18/TAg, DC/IL-12/TAg, DC/IL-18 + IL-12/TAg), stimulated with MC38 tumor cell lysate (2 × 10^6^ cells/mice) or control cells (DC/TAg, DC/EGFP/TAg). On the 29th day, the therapeutic effect of the administration of vaccines was determined. The therapeutic effect of the treatment was evaluated using tumor growth inhibition (TGI), calculated according to the formula: TGI [%] = 100 − (TV_t_/TV_nt_ × 100), where TV_t_ is the median tumor volume in the treated group of mice and TV_nt_ is the median tumor volume in the nontreated group of mice.

### 2.17. Statistics

All the data were analyzed using the GraphPad Prism 8 software (GraphPad Software, Inc.). The cytometric data presentations were prepared using the NovoExpress software. The statistical significance was evaluated using the parametric one-way ANOVA followed by Tukey's multiple comparison post hoc test (data consistent with a Gaussian distribution and equal SD values) or the parametric Brown-Forsythe and Welch ANOVA test followed by Dunnett's T3 multiple comparison post hoc test (data consistent with a Gaussian distribution and not equal SD values) or the nonparametric Kruskal-Wallis test for multiple independent groups followed by Dunn's multiple comparison post hoc test (data inconsistent with a Gaussian distribution). Differences with a *p* value < 0.05 were regarded as significant.

## 3. Results

### 3.1. Efficiency of Dendritic Cell Transduction with Lentiviral Vectors Carrying Sequences of il18 and/or il12 Genes

In the first stage of our research, we determined the effectiveness of bone marrow-derived DC transduction (Figure 1(a)) with lentiviral vectors carrying sequences of *il12* genes and/or the *il18* gene (DC/IL-12, DC/IL-18, DC/IL-18/IL-12). DCs transduced with lentiviral vectors carrying sequences of the enhanced green fluorescent protein (EGFP) gene (DC/EGFP) were used as a transduction control. All types of cells were stimulated with tumor antigens (TAg), and therefore, nontransduced DCs (DC/TAg) were used as a control. The transduction efficiency, phenotype characteristics, and antitumor activity were determined in both *in vitro* and *in vivo* conditions.

The efficiency of DC transduction was determined based on IL-12 and IL-18 concentrations in supernatants obtained from DC cultures (Figure 1(c)) and on the level of mRNA expression for *il18* and *il12p35* genes in these cells (Figure 1(d)). The increased concentration of IL-12 in the supernatants from the above DCs transduced with the *il12* genes alone (DC/IL-12/TAg) or in combination with the *il18* gene (DC/IL-18 + IL-12/TAg, DC/IL-18/IL-12/TAg) correlated with increased relative expression of the *il12p35* gene in these cells. Nevertheless, the highest IL-12 production and *il12p35* gene expression were observed in the DC/IL-12/TAg cells. In the case of DCs transduced with the *il18* gene alone (DC/IL-18/TAg) and in combination with IL-12 (DC/IL-18 + IL-12/TAg, DC/IL-18/IL-12/TAg), increased production of IL-18 was observed in DC/IL-18/TAg and DC/IL-18 + IL-12/TAg, but not DC/IL-18/IL-12/TAg cell types. It was correlated with increased expression of the *il18* gene in these cells. The DC/IL-18/TAg cells were characterized by the highest IL-18 production and expression of the *il18* gene. Nevertheless, dendritic cells transduced with two vectors (DC/IL-18 + IL-12/TAg) showed greater expression and production of IL-12 and IL-18 than DC transduced with a vector carrying both cytokine genes (DC/IL-18/IL-12/TAg). An additional parameter enabling the evaluation of the transduction efficiency of the vaccine cells was the analysis of the expression level of the enhanced green fluorescence (EGFP) protein in control cells (DC/EGFP/TAg) as compared to nontransduced cells (DC/TAg). Analysis of EGFP expression in DC/EGFP/TAg cells by flow cytometry showed high transduction efficiency (99.9%) after introducing a defined number of viral particles into the dendritic cells. Since all types of vectors were introduced into the cells in the same amounts and based on EGFP expression in the control cells, it can be concluded that the obtained vaccine cells account for approximately 71% of the cells transduced for cytokine production (Figure 1(b)).

Subsequent phenotype analysis showed that the type of introduced gene considerably affected the maturation of DCs. Scheme of phenotype analysis of vaccine cells is presented in Figure 1(e). It was shown that, on the 8th day of bone marrow cell culture, more than 70% of the cells expressed CD11c^+^ phenotype (Figure 1(f)). An increase in the expression of MHCII and CD86 on the surface of DCs was related to their contact with lentiviral vectors. However, the introduction of *il12* genes (DC/IL-12/TAg) alone or in combination with the *il18* gene (DC/IL-18 + IL-12/TAg, DC/IL-18/IL-12/TAg) caused further significant elevation of the percentage of MHCII^+^ and CD86^+^ cells in the culture. The transduction of dendritic cells with the Il-18 gene alone did not increase the percentage of MHCII^+^ and CD86^+^ cells. Slight differences in the expression of the CD80 costimulatory molecule on the surface of transduced DCs compared to DC/TAg were observed. The highest percentage of CD80^+^ cells was found in DC/EGFP/TAg culture and the lowest one in DC/IL-18/TAg culture (Figure 1(g)).

The gathered data indicate that the highest maturity stage is shown by DCs simultaneously producing IL-18 and IL-12. Enhanced IL-12 and IL-18 production by transduced DCs and increased *iL12* and *iL18* gene expression in DC/IL-18 + IL-12/TAg cells were considered sufficient for the preparation of a DC-based vaccine. This suggests that such cells may induce a more efficient antitumor immune response.

### 3.2. The Ability of Genetically Modified DCs to Induce a Specific Cellular Response

**Figure 4:**
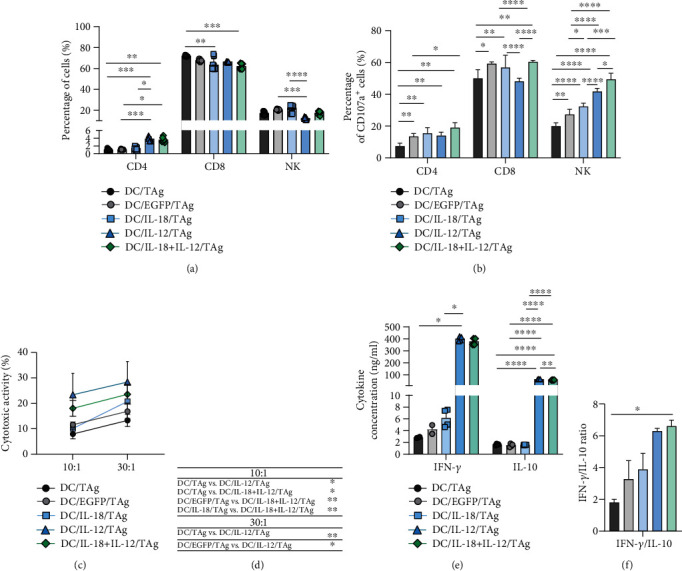
Splenocyte activity after primary stimulation with genetically modified DCs. (a) Phenotypic characteristics of spleen cells obtained after 5-day coculture with genetically modified DCs. (b) The percentage of effector cells (CD107a^+^) was determined among CD4^+^, CD8^+^ T cells, and NK cells after 2-hour incubation with MC38 cells using flow cytometry. (c) The cytotoxic activity of splenic effector cells is shown as the percentage of dead MC38 cells after 4-hour incubation with effector cells in a ratio of 10 : 1 and 30 : 1 E : T (effector cells : MC38/0 target cells). The specific killing of DiO and PI-labeled MC38 tumor cells was performed by flow cytometry. (d) Table of statistically significant changes in the cytotoxic activity of effector splenocytes in a ratio of 10 : 1 and 30 : 1 E : T, respectively. (e) Concentration of IFN-*γ* and IL-10 in supernatants collected after 5 days of coculture of genetically modified DCs with splenocytes was determined using ELISA. (f) The obtained results were additionally presented as the ratio of IFN-*γ* and IL-10 concentrations. Results are presented as mean + SD calculated for at least three repeats in two independent experiments. Differences between groups were estimated using the nonparametric Kruskal-Wallis test followed by Dunn's multiple comparison post hoc test ((a); (c) ratio 30 : 1; (e) concentration of IFN-*γ*; (f)) or the parametric Brown-Forsythe and Welch ANOVA test followed by Dunnett's T3 multiple comparison post hoc test ((b); (c) ratio 10 : 1; (e) concentration of IL-10) (^∗^*p* < 0.05, ^∗∗^*p* < 0.01, ^∗∗∗^*p* < 0.001, ^∗∗∗∗^*p* < 0.0001).

In further in vitro studies, the ability of genetically modified DCs to primarily stimulate naïve T cells and induce a specific antitumor response was evaluated. For this purpose, a 5-day coculture of transduced DCs with splenocytes isolated from the spleens obtained from healthy mice was performed. After this time, we analyzed the changes in the percentages of CD4^+^, CD8^+^ T cells, and NK cells as well as in the percentage of cells expressing CD107a molecules on their surface. Moreover, we investigated the cytotoxic activity of stimulated splenocytes against MC38 cells and their ability to produce IFN-*γ* and IL-10 (Figure 4).

Phenotypic analysis of primed splenocytes from mixed culture showed a significant increase in the percentage of CD4^+^ T lymphocytes after stimulation with DC/IL-12/TAg alone or in combination with IL-18 compared to splenocytes cultured with DC/EGFP/TAg and DC/TAg. The presence of IL-18 alone in such mixed culture did not cause changes in the percentage of CD4^+^ T cells among splenocytes. However, these cells were characterized by an increased ability to secrete cytolytic granules (CD107a, approx. 15-20%) compared to the nontransduced DC group (approx. 7%). Additionally, in consequence of coculture with DC/IL-18 + IL-12/TAg, CD4^+^ T cells demonstrated the greatest capacity to release cytolytic granules. Cytotoxic CD8^+^ T lymphocytes were the largest population in coculture of splenocytes and DCs (approx. 70%). Additionally, approximately 60% of these cells were able to secrete cytolytic granules (showed CD107a expression). We noted a slight decrease in the percentage of CD8^+^ splenocytes cocultured with all types of transduced DCs compared to DC/TAg. Nevertheless, CD8^+^ T cells from coculture with DC/EGFP/TAg, DC/IL-18/TAg, and DC/IL-18 + IL-12/TAg were characterized by increased ability to secrete cytolytic granules. The percentage of NK cells in the mixed cultures was about 20% and did not vary under the influence of DCs transduced for cytokine production. However, a significant increase in the percentage of CD107a^+^NK cells was observed in all groups stimulated with transduced DCs compared to the control group. The greatest ability of splenocytes to release cytolytic granules was observed after stimulation by DC/IL-18 + IL-12/TAg (Figures 4(a) and 4(b)). Phenotypic analysis of primed splenocytes from a mixed culture with DC unstimulated with tumor antigens do not show a significant increase in the percentage of CD4^+^, CD4^+^ T cells, and NK cells as well as in the percentage of cells expressing CD107a molecules on their surface (Supplementary Figure 4). This observation allows to conclude that the immune response generated by DC/TAg is antigen-specific.

Splenocytes stimulated with DC/IL-12/TAg or DC/IL-18 + IL-12/TAg also revealed significantly higher cytotoxic activity toward MC38 than splenocytes from the other groups (Figures 4(c) and 4(d)). Furthermore, in the supernatants from the coculture of splenocytes and DC/IL-12/TAg, increased IFN-*γ* and IL-10 concentrations compared to supernatants from the coculture with DC/TAg were observed. The calculated IFN-*γ*/IL-10 ratio indicates that DC/IL-18 + IL-12/TAg considerably increased the capacity of splenocytes for IFN-*γ* production (Figures 4(e) and 4(f)).

The obtained data indicate that the applied transduction method allows one to obtain mature and active bone marrow-derived DCs with overproduction of IL-18 and/or IL-12. Moreover, DCs simultaneously producing IL-12 and IL-18 may be a potential component of anticancer therapy.

### 3.3. The Ability of Modified DC-Based Vaccines to Migrate to the Tumor-Draining Lymph Nodes and Infiltrate Tumor Tissue of MC38 Murine Colon Carcinoma

Mice with established tumors were treated peritumorally (p.t.) with DCs genetically modified for the production of IL-18 and/or IL-12 and control cells, stimulated with TAg. Tumor-draining lymph nodes, spleens, and tumors were collected from mice on the 3rd, 5th, and 7th days after a single administration of DC-based vaccines (Figure 2(a)) to estimate changes in the activation of the local and systemic antitumor response.

To identify transduced cells in lymph nodes and tumor tissue, DCs were stained with the fluorescent dye CFDA-SE before injection. The intensity of fluorescence of CFDA-SE labeled cells was analyzed using flow cytometry. All vaccine cells showed a similar and detectable level throughout the in vitro culture and in vivo experiment (on the 3rd, 5th, and 7th days after injection) (Figure 2(b)). CFDA-SE labeled cells were identified in tumor tissue and lymph nodes applying the analysis scheme presented in Figure 2(c). The total number of CFDA-SE-labeled DCs in tissues was measured using counting beads. The table shows the percentages of CFDA-SE-labeled DCs identified in tumor tissue (TT) and tumor-draining lymph nodes (tLNs) on the 3rd, 5th, and 7th days after injection (Figure 2(d)).

The graph presents the mean number of DCs per gram of tumor nodule. The most effective infiltration of all modified DCs except DC/EGFP/TAg was observed on the 3rd day after injection, and the number of these cells decreased over the following days. In the case of DC/EGFP/TAg, the highest numbers of the cells were identified in the tumor tissues on the 5th day after vaccination. It should be emphasized that DC/IL-18 + IL-12/TAg cells showed the greatest ability to infiltrate tumor tissue already on the 3rd day after administration, and then, their number during the following days of observation diminished more slowly than in other groups (Figures 2(c) and 2(e)).

Determination of vaccine cells' ability to migrate to the lymph nodes was also presented as the mean number of DCs per 1 million isolated lymph node cells (Figures 2(c) and 2(f)). The highest number of peritumorally injected cells in the lymph nodes on the 3rd, 5th, and 7th days after injection was noted in the group of mice vaccinated with DC/TAg, while DC/IL-12/TAg was characterized by the lowest ability to migrate to the lymph nodes among all types of vaccine cells.

The numbers of CFDA-SE^+^ DCs identified in tumor tissue and lymph nodes depended on the type of applied vaccine cells and the time since DC administration. The efficiency of DC migration to tLNs is strictly dependent on the degree of their moderate maturity. The most mature DCs lose the ability to migrate to tLNs, but infiltrate the tumor nodule more efficiently, thus changing the cytokine balance in the TME.

### 3.4. Influence of Genetically Modified DCs on Induction of Local Antitumor Response

#### 3.4.1. In Tumor Tissue

**Figure 5:**
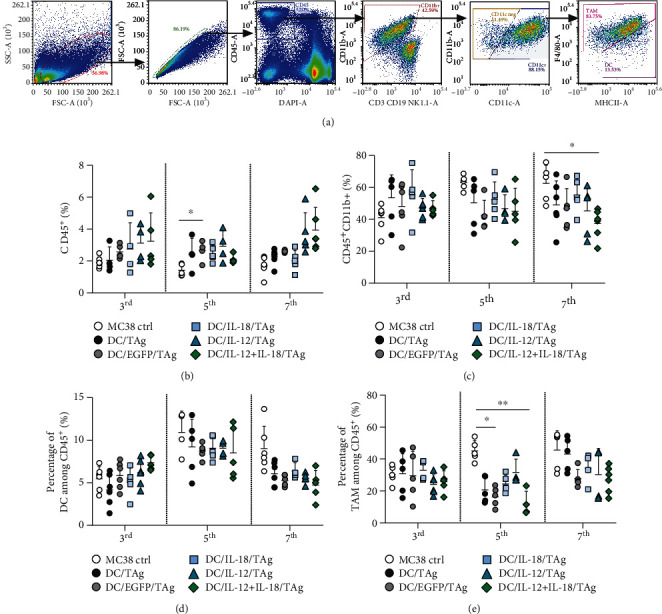
Evaluation of myeloid cell subpopulation infiltrating MC38 tumor tissue after administration of DC-based vaccines. (a) Scheme of the multiparameter flow cytometric analysis of DC and TAM in MC38 tumor nodules. The analysis was started by eliminating debris and cell aggregates; then, live leukocytes (DAPI^−^CD45^+^) were gated, and among them, CD11b^+^ myeloid cells were identified. The CD11b^+^ cells were subsequently separated for expression of the CD11c^+^ marker. Among the CD11c^+^ cells, the DC (CD11c^+^F4/80^int^MHCII^+^) and TAM (CD11c^+^F4/80^+^) population were identified. (b) Percentage of CD45^+^ cells in tumor tissue. (c) Percentage of CD45^+^CD11b^+^ cells in tumor tissue. (d) Percentage of DC infiltrating tumor tissue. (e) Percentage of TAM in tumor tissue. Results are presented as mean ± SD calculated for 5-6 mice per group. Differences between groups were estimated using the nonparametric Kruskal-Wallis test followed by Dunn's multiple comparison post hoc test ((e) percentage of TAM in tumor tissue on the 5th day), the parametric one-way ANOVA followed by Tukey's multiple comparison post hoc test ((c) percentage of CD45^+^CD11b^+^ on the 7th day), or the parametric Brown-Forsythe and Welch ANOVA test followed by Dunnett's T3 multiple comparison post hoc test ((b) percentage of CD45^+^ cells on the 5th day) (^∗^*p* < 0.05, ^∗∗^*p* < 0.01).

To determine changes in the percentage and activity of immunocompetent cell infiltrating tumors, the multiparameter flow cytometry method was applied. Myeloid cell subpopulations were identified applying the analysis scheme presented in Figure 5(a).

An increased influx of leukocytes (CD45^+^ cells) into tumor tissues compared to MC38 control was observed after treatment of mice with DC-based vaccines. The largest but not statistically significant influx of CD45^+^ cells was found after administration of DC/IL-18 + IL-12/TAg cells on the 3rd and 7th days, whereas the highest elevation of immune cells after administration of DC/IL-12/TAg was observed on the 5th day (Figure 5(b)).

In the case of CD11b^+^ cells, the most visible differences in the percentage of cells infiltrating tumor tissue were observed between DC-vaccinated mouse groups and the untreated mice. After the application of DC-based vaccines, decreasing percentages of CD11b^+^ cells were observed. However, only on the 7th day after application of DC/IL-18 + IL-12/TAg, a significantly lower percentage of CD11b^+^ cells compared to the MC38 control group was observed (Figure 5(c)).

The obtained data also revealed slight changes in the percentages of DCs (CD11b^+^CD11c^+^F4/80int) infiltrating the tumor tissues in the aftermath of DC injection. Although the difference was not statistically significant, we observed a higher percentage of these cells in the group of mice treated with DC/EGFP/TAg, DC/IL-12/TAg, and DC/IL-18-IL-12/TAg than in the MC38 control group just on the 3rd day after administration. The highest influx of DCs on the 3rd day was observed after administration of DC/IL-18 + IL-12/TAg. It should be emphasized that the DC/IL-18 + IL-12/TAg vaccine cells in the tumor tissue were estimated at 0.07%. In contrast, DC infiltrating tumor tissue accounted for approx. 5-11% among CD45^+^ cells. On the 5th day, we observed an increase in the influx of DC into the tumor tissues in all groups compared to the 3rd day. However, the percentage of DCs after DC-based vaccination was lower than in the MC38 control group. On the last day of the analysis, the percentage of DCs in all examined groups decreased, without any significant differences between groups inoculated with DC-based vaccines (Figure 5(d)).

More visible fluctuations were observed in the percentage of tumor-associated macrophage (TAM, CD11b^+^CD11c^+^F4/80^+^) infiltrating tumors. Although on the 3rd day after administration of DC-based vaccines, no differences in the percentage of TAM were observed; on the 5th day after injection, significant decreases of percentages of TAM in the DC/EGFP/TAg and DC/IL-18 + IL-12/TAg groups were observed compared to the MC38 control group. The same trends, but not statistically significant, on the 7th day were noted (Figure 5(e)).

**Figure 6:**
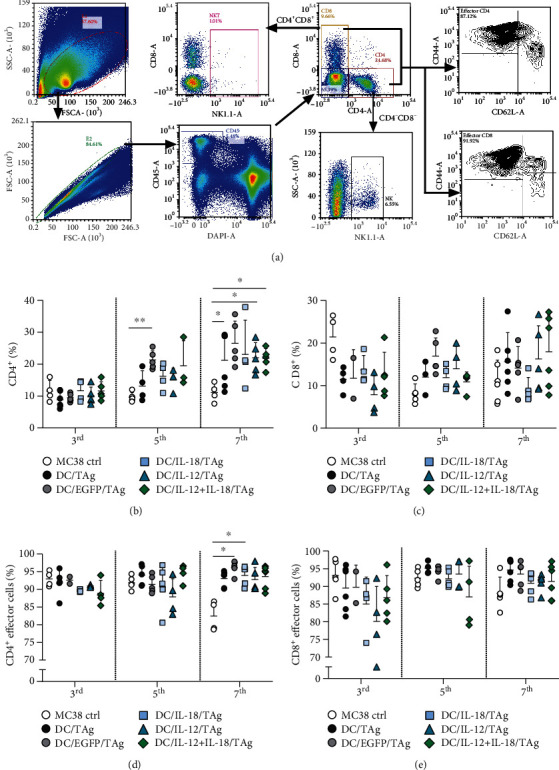
Evaluation of lymphoid cell subpopulation infiltrating the tumor tissue after administration of DC-based vaccines. (a) Scheme of the multiparameter flow cytometric analysis of CD4^+^ and CD8^+^ T cells, NK and NKT cells, and T lymphocyte activity. The analysis was started by eliminating debris and cell aggregates; then, live leukocytes (DAPI^−^CD45^+^) were gated, including CD4^+^ T cells and CD8^+^ T cells. NKT cells were identified among CD4^+^CD8^+^ cells, while NK cells were extracted from the CD4-CD8- cells, based on NK1.1 marker expression. Effector T lymphocytes were defined as CD44^+^ CD62L^−^. (b, c) Percentage of CD4^+^ and CD8^+^ T lymphocytes among CD45^+^ cells in tumors on the 3rd, 5th, and 7th days after DC injection. (d, e) Percentage of effector cells among CD4^+^ and CD8^+^ T cells in tumors. Results are presented as mean ± SD calculated for 5-6 mice per group. Differences between groups were estimated using the parametric Brown-Forsythe and Welch ANOVA test followed by Dunnett's T3 multiple comparison post hoc test ((b) percentage of CD4^+^ on the 5th and 7th days; (d) percentage of CD4^+^ effector cells on the 7th day) (^∗^*p* < 0.05, ^∗∗^*p* < 0.01).

The aim of the second part of the multicolor flow cytometric analysis was to evaluate the lymphoid cell subpopulation (CD4^+^, CD8^+^ T lymphocytes, NK cells, NKT cells), infiltrating the tumor tissue on the 3rd, 5th, and 7th days after vaccine administration. Based on the expression of CD44 and CD62L markers, effector cells (CD44^+^ CD62L-) among CD4^+^ and CD8^+^ T lymphocytes were identified (Figure 6(a)).

On the 3rd day after treatment with the DC-based vaccine, no changes in the percentage of CD4^+^ in the tumor tissue collected from the experimental mice were observed (Figure 6(b)). On the other hand, the percentage of CD8^+^ lymphocytes in tumor decreased in the aftermath of DC-based vaccine injection (Figure 6(c)). Nevertheless, the percentage of CD4^+^ and CD8^+^ effector cells was slightly lower in the groups receiving DC vaccines producing cytokine than in the untreated group (Figures 6(d) and 6(e)). Furthermore, on the 5th day after injection, an increase in the percentage of CD4^+^ and CD8^+^ cells in tumor tissue from mice treated with DC-based vaccines was observed. We found the highest percentage of CD4^+^ and CD8^+^ cells after administration of DC/EGFP/TAg (Figures 6(b) and 6(c)) and a similarly high percentage of CD4^+^ cells but the lowest percentage of CD8^+^ cells in the group treated with DC/IL-18 + IL-12/TAg. Meanwhile, no visible differences in the percentage of both types of effector cells were noted (Figures 6(d) and 6(e)).

Importantly, the substantial changes in the percentage of lymphoid cells in the tumor tissue collected from the experimental mice were observed just on the 7th day. A significantly higher percentage of CD4^+^ cells in the group of mice treated with DC/EGFP/TAg, DC/IL-12/TAg, and DC/IL-18 + IL-12/TAg compared to untreated mice was found (Figure 6(b)). Although statistically significant differences in CD8^+^ percentage on the 7th day were not observed, it seems that DC/IL-18 + IL-12/TAg cells also induced the highest infiltration of CD8^+^ cells compared to the MC38 control group (Figure 6(c)). On that day, the greatest influx of CD4^+^ and CD8^+^ effector cells into the tumor tissue after DC-based vaccines administration was observed (Figures 6(d) and 6(e)).

No significant changes in the percentage of NK cells in tumor tissue were noted. On the 3th and 5th days after administration of DC-based vaccines, a slight increase in the percentage of NK cells was observed in the tumor tissue, but this effect was not observed on the 7th day after vaccination (Supplementary Figure 1A). However, a significantly higher percentage of NKT cells was observed in the group of mice treated with DC/IL-18/TAg on the 3rd day after DC-based vaccine administration. At the other time point of the analysis, no changes in the percentage of NKT cells were observed (Supplementary Figure 1B).

In conclusion, the presented data suggest that, only on the 7th day after a single peritumoral administration of DC-based vaccines, we can observe substantial changes in the percentage of cell CD45^+^ cell infiltrating tumor tissue. Tumor tissue obtained from mice treated with DC/IL-18 + IL-12/TAg revealed a significantly lower percentage of CD11b^+^ cells, and therein thus, the percentage of DC and TAM was observed. In contrast, an increased percentage of CD4^+^ and CD8^+^ T cells infiltrating the tumor tissue after administration of these vaccines was noted.

#### 3.4.2. In Tumor-Draining Lymph Nodes

**Figure 7:**
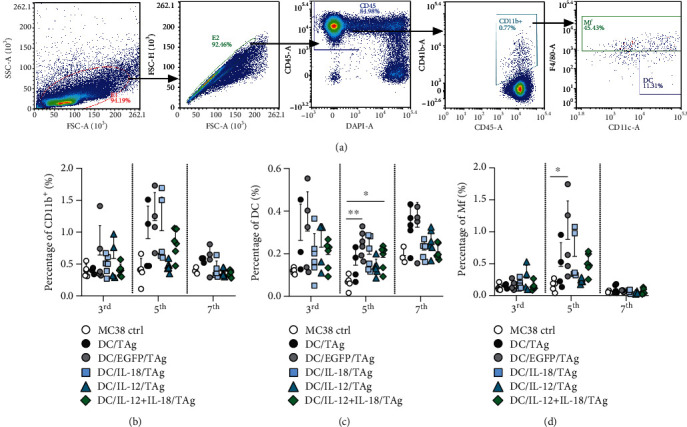
Evaluation of myeloid cell subpopulation in tumor-draining lymph nodes after administration of vaccine cells. (a) Scheme of the multiparameter flow cytometric analysis of DC and Mf on the 3rd, 5th, and 7th days after single injection of DCs in tumor-draining lymph nodes. The analysis was started by eliminating debris and cell aggregates; then, live leukocytes (DAPI^−^CD45^+^) were gated, and among them, CD11b^+^ myeloid cells were identified. The CD11b^+^ cells were subsequently separated for expression of the CD11c^+^ marker. Among the CD11c^+^ cells, the DC (CD11c^+^F4/80int) and TAM (CD11c^+^F4/80^+^) populations were identified. (b) Percentage of CD11b^+^ cells in lymph nodes. (c) Percentage of DCs in lymph nodes. (d) Percentage of Mf in lymph nodes. Results are presented as mean + SD calculated for 5-6 mice per group. Differences between groups were estimated using the parametric Brown-Forsythe and Welch ANOVA test followed by Dunnett's T3 multiple comparison post hoc test ((c) percentage of DC cells on the 5th day) or the nonparametric Kruskal-Wallis test followed by Dunn's multiple comparison post hoc test ((d) percentage of Mf on the 5th day) (^∗^*p* < 0.05, ^∗∗^*p* < 0.01).

In order to evaluate the effect of DC-based vaccines on the development of an antitumor response in tumor-draining lymph nodes, changes in the percentages of myeloid and lymphoid cells were analyzed. First, the impact of a single administration of DCs on differences in the percentage of the myeloid cell population (CD11b^+^) in the tumor-draining lymph nodes on the 3rd, 5th, and 7th days using flow cytometry was assessed (Figure 7(a)). We observed alterations in the percentage of CD11b^+^ cells that were dependent on both time point and type of administered vaccine. However, the evident increase in the percentage of these cells was dependent on the increase in the percentage of macrophages (Mf) (Figure 7(b)).

On the other hand, the highest CD11b^+^ cell percentage was noted after treatment with DC/EGFP/TAg on each day of analysis. Estimation of the DC percentage showed that these cells represented only a small population of myeloid cells in tumor-draining lymph nodes (Figure 7(c)). However, on the 5th day after treatment, the percentage of host DCs declined compared to the whole CD11b^+^ population. In contrast, an increase in the percentage of Mf was observed exactly on the 5th day after cell vaccine administration. Moreover, the percentage of Mf was significantly elevated in the group of mice treated with DC/EGFP/TAg compared to the control only on this day. On the 7th day of the analysis, the percentage of Mf in all groups was negligible (Figure 7(d)).

**Figure 8:**
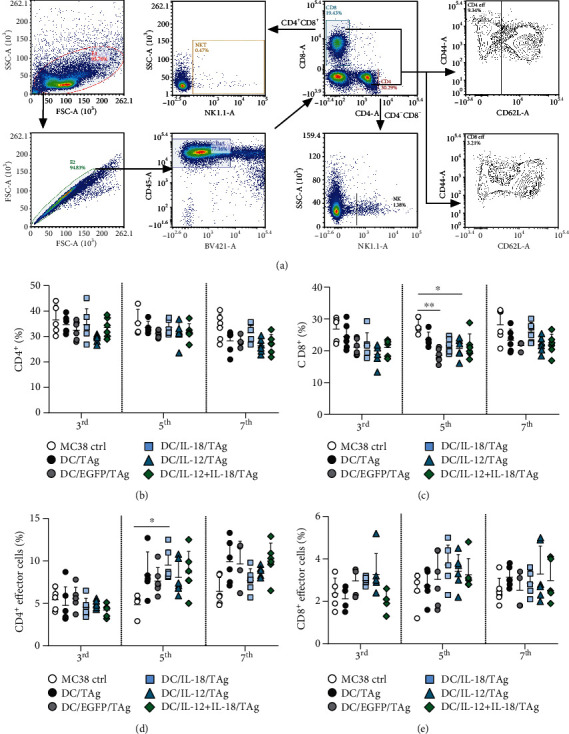
Evaluation of CD4^+^and CD8^+^ T lymphocytes and their activity in tumor-draining lymph nodes after administration of vaccine cells. (a) Scheme of the multiparameter flow cytometric analysis of CD4^+^ and CD8^+^ T cell, NK and NKT cell, and T lymphocyte activity on the 3rd, 5th, and 7th days after single injection of DCs in tumor-draining lymph nodes. The analysis was started by eliminating debris and cell aggregates; then, live leukocytes (DAPI^−^CD45^+^) were gated, including CD4^+^ T cells and CD8^+^ T cells. NKT cells were identified among CD4^+^CD8^+^ cells, while NK cells were extracted from the CD4-CD8- cells, based on NK1.1 marker expression. Effector T lymphocytes were defined as CD44^+^ CD62L^−^. (b) Percentage of CD4^+^ T lymphocytes in lymph nodes. (c) Percentage of CD8^+^ T lymphocytes in lymph nodes. (d) Percentage of effector cells among CD4^+^ T cells in lymph nodes. (e) Percentage of effector cells among CD8^+^ T cells in lymph nodes. Results are presented as mean + SD calculated for 5-6 mice per group. The differences between the groups were estimated using the parametric Brown-Forsythe and Welch ANOVA test followed by Dunnett's T3 multiple comparison post hoc test (^∗^*p* < 0.05, ^∗∗^*p* < 0.01).

In contrast to myeloid cell analysis (Figure 8(a)), no significant changes in the percentage of CD4^+^, CD8^+^, NK, and NKT cells after multiparameter cytometric analysis of lymph nodes (analogous to tumor tissue analysis) were revealed (Figures 8(b) and 8(c), Supplementary Figure 2).

The relations among particular groups of effector cells appeared to be much more diverse. No differences in the percentage of effector CD4^+^ cells on the 3rd day after DC-based vaccine administration were detected (Figure 8(d)), but on the 5th and 7th days, we found an increase in the percentage of these cells in each group of mice treated with DC-based vaccines. The highest percentage of effector CD4^+^ cells was noted after administration of DC/IL-18/TAg on these days. The increased percentage of effector CD4^+^ cells in all therapeutic groups was also maintained on the 7th day of analysis and was the highest in the DC/IL-18 + IL-12/TAg group.

Importantly, the analysis of CD8^+^ effector cells showed a higher percentage of these cells in the group of mice treated with DC/EGFP/TAg, DC/IL-18/TAg, and DC/IL-12/TAg on the 3rd day after treatment (Figure 8(e)). On the 5th day, an increase in the percentage of CD8^+^ effector cells was observed in all groups treated with DC-based vaccines. The highest percentage of these cells was detected in the groups of mice treated with DCs transduced to produce cytokines. The increased percentage of CD8^+^ effector cells after administration of DCs producing IL-18 and/or IL-12 persisted until the 7th day.

In conclusion, the analysis of the size of the myeloid and lymphoid cell population in the lymph nodes showed that a single administration of DC-based vaccines did not considerably influence the changes in percentages of defined cell subpopulations. DC/EGFP/TAg vaccines caused only an increase in the percentage of myeloid cells (CD11b^+^), both DC and Mf. Meanwhile, DCs producing IL-18 and/or IL-12 were able to induce activation of CD4^+^ and CD8^+^ cells on the 5th day after application, and this effect persisted until the 7th day of the experiment. Based on these results, we can conclude that DCs transduced for the simultaneous production of IL-18 and IL-12 can induce local antitumor responses, already on the 5th day after the administration of vaccines.

### 3.5. Influence of Genetically Modified DCs on Induction of a Systemic Antitumor Response

**Figure 9:**
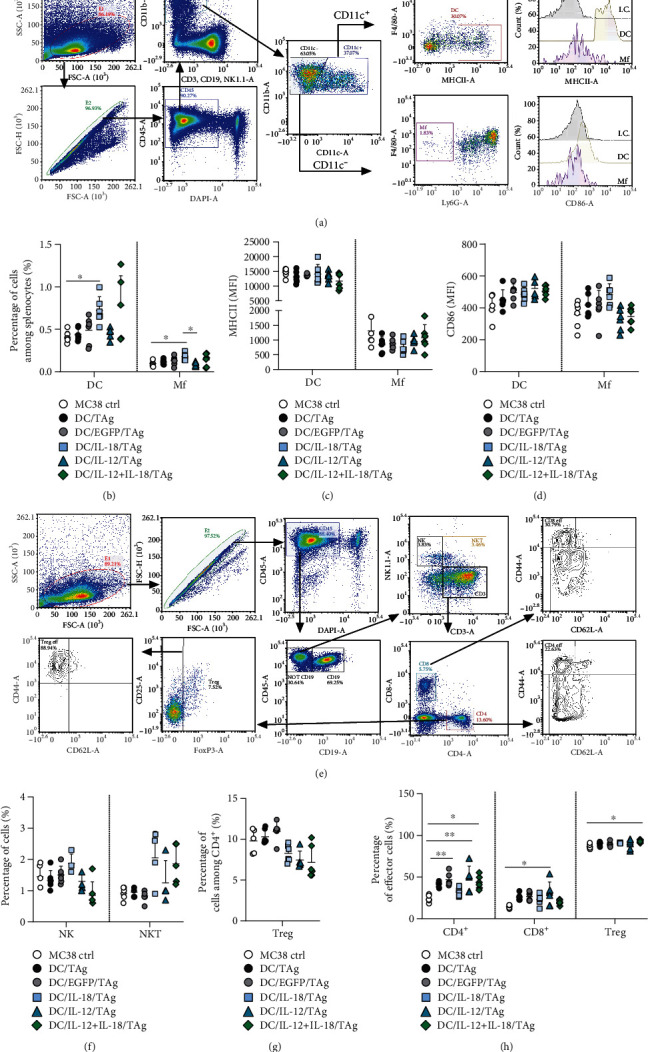
Effect of administration of transduced DCs on myeloid and lymphoid cell populations in the spleen on the 7th day after injection. (a) Scheme of multiparameter flow cytometric analysis of DCs and Mf on the 7th day after single injection of DCs in the spleen. (b) Percentage of DCs and Mf in the spleen. (c, d) Expression of MHC II and CD86 molecules on the surface of DCs and Mf in the spleen. (e) Scheme of multiparameter flow cytometric analysis of CD4^+^, CD8^+^, NK, and NKT on the 7th day after single injection of DCs in the spleen. (f) Percentage of NK and NKT cells. (g) Percentage of Treg cells among CD4^+^ T lymphocytes. (h) Percentage of effector cells among CD4^+^, CD8^+^, and Treg cells. Results are presented as mean + SD calculated for 5-6 mice per group. Differences between groups were estimated using the parametric Brown-Forsythe and Welch ANOVA test followed by Dunnett's T3 multiple comparison post hoc test (^∗^*p* < 0.05).

In the next stage of the experiments, in a manner similar to the study of tumor tissue and lymph nodes, we performed extended flow cytometric analyses of myeloid and lymphoid cells among splenocytes obtained from mice on the 7th day after treatment with DC-based vaccines. During myeloid cell analysis, among live leukocytes (DAPI^−^CD45^+^) in the spleen, we identified DC (CD11b^+^CD11c^+^F4/80^lo^MHCII^+^) and Mf (CD11b^+^CD11c^−^F4/80^+^) and evaluated the expression of MHCII and CD86 molecules on the surface of these cells (Figure 9(a)).

After administration of DC/IL-18/TAg and DC/IL-18 + IL-12/TAg vaccines, the presence of DCs and Mf in the spleen did not exceed one percent of all leukocytes, although it was higher than in other groups (Figure 9(b)). We noted a slight decrease in the expression of the MHC II molecules on the surface of DCs and Mf compared to the control but no changes among treated groups (Figure 9(c)). In contrast, the expression of CD86 on DCs was upregulated after DC-based vaccine application, but none of these changes was statistically significant. The highest expression of CD86 on DC surfaces was recorded after administration of DC/IL-12/TAg. Meanwhile, after administration of DC/IL-18/TAg, the highest expression of CD86 on the surface of Mf was found (Figure 9(d)).

We examined, in the same manner, lymphoid cells among spleen leukocytes: CD4^+^ T lymphocytes (CD3^+^CD4^+^), CD8^+^ T lymphocytes (CD3^+^CD8^+^), NK (NK1.1^+^), NKT (CD3^+^NK1.1^+^) cells, and Tregs (CD3^+^CD4^+^CD25^+^FoxP3^+^) using the multiparametric flow cytometry analysis protocol. The percentage of effector cells (CD44^+^CD62L^−^) among CD4^+^, CD8^+^, and Treg was also assessed (Figure 9(e)). The application of DC-based vaccines did not contribute to differences in the percentage of CD4^+^ and CD8^+^ T cells among splenocytes compared to the nontreated group (Supplementary Figure 3). However, differences in the percentages of NK and NKT cells were observed (Figure 9(f)).

After administration of DC/IL-18/TAg, the percentage of NK cells was the highest, whereas after treatment with DC/IL-18 + IL-12/TAg, it was the lowest. The application of DCs transduced for cytokine production increased the percentage of NKT cells in the spleen (Figure 9(f)). Moreover, the lowest percentage of Tregs among splenocytes obtained from mice treated with DCs producing IL-18 and/or IL-12 was observed (Figure 9(g)). Administration of DC/IL-18/TAg caused a statistically significant increase in the percentage of effector cells among CD4^+^ and Tregs and a slight increase in the percentage of effector cells among CD8^+^ T cells compared to splenocytes obtained from MC38 control mice (Figure 9(h)).

**Figure 10:**
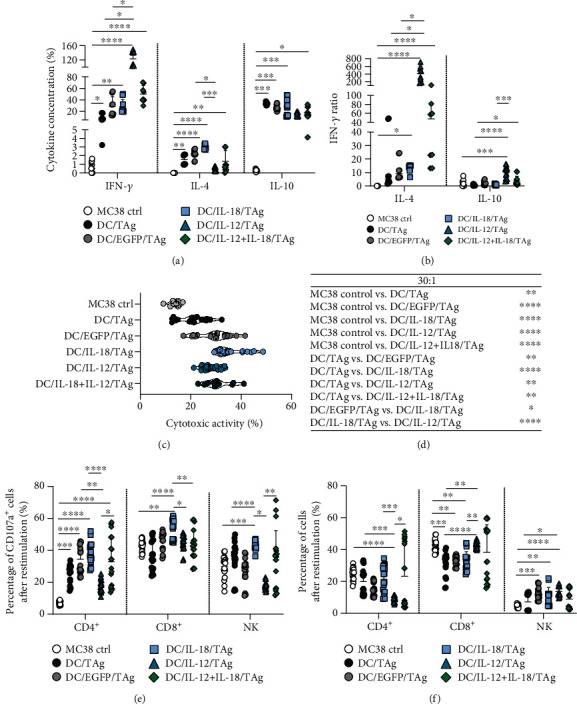
Effect of administration of transduced DCs on induction of antitumor response in the spleen on the 7th day after injection. (a) Cytokine concentration (IFN-*γ*, IL-4, IL-10) in supernatants after restimulation of spleen cells with MC38 cells. (b) Ratio of IFN-*γ* and IL-4 or IL-10 concentration in supernatants after restimulation. (c, d) Cytotoxic activity of splenocytes (effector cells) against DiO^+^ MC38 cells (target cells). (e) Percentage of CD107a^+^ cells among CD4^+^, CD8^+^, and NK cells measured by CD107a degranulation assay. (f) Percentage of CD4^+^, CD8^+^, and NK cells among splenocytes after restimulation. Results are presented as mean ± SD calculated for 5-6 mice per group. Differences between groups were estimated using the nonparametric Kruskal-Wallis test followed by Dunn's multiple comparison post hoc test or the parametric Brown-Forsythe and Welch ANOVA test followed by Dunnett's T3 multiple comparison post hoc test (^∗^*p* < 0.05, ^∗∗^*p* < 0.01, ^∗∗∗^*p* < 0.001, ^∗∗∗∗^*p* < 0.0001).

In the next stage of research, we evaluated the development of a systemic antitumor response after treatment with DC-based vaccines. For this purpose, the splenocytes were restimulated ex vivo with MC38 cells. After 5 days of coculture, the supernatant and cells were collected. The concentration of IFN-*γ*, IL-4, and IL-10 in the supernatants was determined. Cytotoxic activity of restimulated splenocytes towards MC38 cells, their phenotype (CD4^+^, CD8^+^, NK1.1^+^), and expression of CD107a reflecting the catalytic granule release process were estimated. An increase in concentration of the tested cytokines (IFN-*γ*, IL-4, IL-10) in the supernatants from the above mixed culture of MC38 cells and splenocytes obtained from mice treated with DC-based vaccines compared to the MC38 control was observed. After administration of DC/IL-12/TAg, the highest concentration of IFN-*γ* was detected, while the highest concentration of IL-4 was determined in the group of mice treated with DC/IL-18/TAg. The concentration of IL-10 remained at a similar level in all treated group (Figure 10(a)). The highest ratio of IFN-*γ*/IL-4 and IFN-*γ*/IL-10 was calculated for the DC/IL-12/TAg group (Figure 10(b)). It should be noted that all DC-based vaccines induced more efficient cytotoxic activity of restimulated spleen cells towards MC38 tumor cells than splenocytes obtained from MC38 control mice. Furthermore, the administration of the DC/IL-18/TAg vaccine caused the highest cytotoxic activity of splenocytes (Figures 10(c) and 10(d)). This effect correlated with the highest percentage of CD4^+^CD107a^+^, CD8^+^CD107a^+^, and NK1.1^+^CD107a^+^ cells among restimulated splenocytes from the DC/IL-18/TAg-treated mice (Figure 10(e)). The restimulation of splenocytes by MC38 cells confirmed the differential impact of DC-based vaccines on the percentage of CD4^+^, CD8^+^, and NK1.1 cells. Among the restimulated splenocytes, the lowest percentage of CD4^+^ and the highest percentage of CD8^+^ and NK cells after administration of DC/IL-12/TAg were recorded (Figure 10(f)).

In summary, the greatest stimulation of the systemic immune response was observed in the group of mice treated with DC/IL-18/TAg on the 7th day after a single administration of cellular vaccines. A single administration of these cells significantly increased the percentage of DCs and macrophages among splenocytes, which showed increased expression of CD86 molecules on the surface. Furthermore, among splenocytes obtained from mice after DC/IL-18/TAg therapy, we found an increase in the percentage of NK and NKT cells, as well as CD4^+^ and CD8^+^ effector cells, and a decrease in the percentage of Treg cells. Restimulated splenocytes collected from mice after administration of DC/IL-18/TAg showed the highest cytotoxic activity, which correlated with an increased percentage of CD107a^+^ cells among CD4^+^, CD8^+^, and NK cells.

### 3.6. Growth of MC38 Tumor in Immunotherapy-Treated Mice

In the last stage of the presented research, mice with established tumors were treated peritumorally (p.t.) with DCs genetically modified for the production of IL-18 and/or IL-12 and control cells, stimulated with TAg according to the scheme presented in Figure 3(a). The tumor growth inhibition (TGI) calculated on the 29th day of the experiment for all groups of mice indicated the impact of the use of transduced dendritic cells for the cytokine production on growth inhibition of MC38 tumors.

The application of dendritic cells producing IL-12 only resulted in a statistically significant TGI of MC38 tumors amounted to 65.77%, whereas the use in the therapy of dendritic cells producing IL-12 and IL-18 simultaneously resulted in increase in tumor growth inhibition to 70.25%. The use of DC/IL-18/TAg in the therapy resulted in the inhibition of the growth of MC38 tumors by 50.30%. The introduction of the control vector into the vaccine cells did not inhibit tumor growth as compared to the untransduced cells.

These data confirm that the anticancer response initiated by a single administration of IL-12 and/or IL-18 secreting vaccines can be enhanced by repeated administration of these vaccines. Moreover, the increase in the number of administrations revealed the different effects of the DC-based vaccines depending on the type of cytokines which they produce.

## 4. Discussion

The main purpose of our research was to develop and characterize the antitumor activity of a novel cancer vaccine, based on DCs transduced for the simultaneous production of IL-12 and IL-18. Enhancement of the specific antitumor activity of the transduced DCs was gained by stimulation with tumor antigens (TAg).

First of all, we determined the efficiency of bone marrow-derived dendritic cell (DC) transduction for overproduction of IL-18 and/or IL-12 using a 3rd generation lentivirus system. This method is much more efficient than the retroviral vectors used for cell transduction. Another advantage of lentivirus-based delivery systems is that relatively large sequences of genes can be introduced into a target cell, reaching up to 9 kilobase pairs without affecting the vector titer [29]. Thus, along with inducing stable and long-term transgene expression, lentiviral vectors are a powerful tool for the delivery of genetic material into hosts.

We found that the applied transduction method was an efficient tool for the preparation of vaccine cells, which caused high transgene expression as well as high production of IL-12 and IL-18. In our study, supernatants from DCs transduced with lentiviral vectors carrying *il12p70* or *il18* genes after 24 h incubation contained approximately 100-200 pg/ml of IL-12p70 and 100–200 pg/ml of IL-18. Moreover, cytokine production was correlated with increased expression of the *il12p35* and *il18* genes in these cells. Vujanovic with coworkers, who transduced DCs with adenoviral vectors carrying *il12p70* or *il18* genes, observed production of this cytokine at the level of approximately 10-20 ng/ml of IL-12p70 and 200–300 pg/ml IL-18 but after 48 h incubation at 37°C [30]. The prolonged culture of our transduced DCs (to 72 h) resulted in the elevation of IL-12 production to 20 ng/ml, which proves that the obtained cells are capable of long-term (several days) cytokine delivery.

The subsequent step of transduced DCs' characteristics showed essential changes in the increase of MHCII and CD86 expression on the surface of these cells as a result of the introduction of the *il12p70* gene alone and combination with the *il18* gene. On the other hand, transduction with the *il18* gene did not influence the expression of costimulatory molecules on the DC surface. Stober and coauthors reported the effect of exogenous IL-12 alone on increasing the expression of MHC II and CD86 molecules on the surface of DCs and the minor effect of IL-18 stimulation on changes in the expression of these molecules [31]. We observed that the combination of these two cytokines strongly activates DCs. In our research, we only observed that slight differences in the expression of the CD80 costimulatory molecule on the surface of transduced DCs compared to DC/TAg were observed but phenotypic analysis of vaccine cells was performed 24 hours after transduction. According to other reports, expression of the CD86 molecule by DCs is generated shortly after cell activation but remains on the cell surface for a relatively short time (48 h). APC activation results in maximal expression of the CD86 molecule as early as 6-8 hours after stimulation. In contrast, expression of the CD80 molecule by DCs is induced slower, but its expression on the surface of DC cells is longer (4-5 days). CD80 particles appear on the cell surface only 24-48 hours after the stimulation of the tested APCs. The phenotypic analysis performed 24 hours after transduction allowed to show changes only in the expression of the CD86 molecule on the DC surface, because this molecule is generated in a short time. However, no significant changes were observed in the expression of the CD80 molecule, which is induced on the DC surface more slowly [32]. In our study, we found that in the case of DCs transduced with lentiviral vector changes in the expression of MHC II and costimulatory molecules were induced not only by incorporation of cytokine genes but also by the presence of lentiviral molecules, as was observed for DC/EGFP/TAg. Literature data show that lentiviral vectors can stimulate DCs through TLR3 and TLR7 receptors, which leads to their maturation and increased expression of MHC II and costimulatory molecules [33].

To confirm that phenotypic and functional changes in transduced DCs significantly affect the ability of transduced DCs to activate naive T cells and induce an antitumor response, the cytotoxic activity of splenocytes cultured in the presence of genetically modified DCs stimulated with MC38 tumor antigens was determined. We found that the presence of DC/IL-18 + IL-12/TAg induced an increase in cytotoxicity of spleen cells against MC38 cancer cells. Analysis of the mechanism of cytotoxicity used by effector T lymphocytes and NK cells revealed that CD4^+^ and CD8^+^ T lymphocytes and NK cells stimulated with DC/IL-18 + IL-12/TAg indicated a greater ability to secrete cytolytic granules than lymphocytes cultured in the presence of DC/TAg or DC/EGFP/TAg. Moreover, splenocytes in a mixed culture with DC/IL-18 + IL-12/TAg showed higher potential for IFN-*γ* production compared to DC/TAg stimulated splenocytes. The obtained results confirm that the influence of IL-18 and IL-12 on splenocytes is closely related. Many studies have demonstrated the synergistic effect of IL-12 and IL-18 on NK cells. For instance, Chaix et al. reported that NK stimulation with IL-18 is necessary for IFN-*γ* production by these cells in response to IL-12 [34]. Likewise, Martinović et al. observed that simultaneous stimulation of IL-18 and IL-12 induced greater NK cell degranulation and IFN-*γ* production than stimulation with IL-18 or IL-12 independently [35]. Okamoto et al. reported the synergistic effect of IL-18 and IL-12 on Th1, CD4^+^, and CD8^+^ effector T cells just over 20 years ago [36]. They confirmed that IL-18 efficiently induces the development of type I CD8^+^ effector T cells with strong cytotoxic activity. In contrast, IL-12 suppressed the maturation of CD8^+^ T cells to IFN-*γ* producing CTLs, whereas it promoted the maturation of CD4^+^ T effector cells producing IFN-*γ*. However, when both cytokines were acting together, they induced the development of type I CD8^+^ effector T cells more efficiently [36]. Other researchers found that IL-18 and IL-12 synergistically induced IL-8 production by periphery blood-derived NK cells [37]. IL-12p70 and IL-18 cooperate to promote secretion of IFN-*γ* by Th1 cells and foster the proliferation of CD4^+^ T effector cells. IL-12p70 induces expression of the IL-18 receptor (IL-18R) on the surface of naïve T cells, whereas IL-18 potentiates the differentiation of Th1 cells initiated by IL-12p70 [30]. All conducted *in vitro* studies and literature reports confirm that DCs simultaneously producing IL-12 and IL-18 may be a potential component of anticancer therapy.

An important stage of our research was to determine the genetically modified DC cell's ability to migrate to the tumor-draining lymph nodes and infiltrate the tumor tissue of MC38 murine colon carcinoma. For this purpose, mice with established tumors were peritumorally (p.t.) administered DC-based vaccines. DCs genetically modified for the production of IL-18 and/or IL-12 and stimulated with TAg, and control cells were identified in lymph nodes and tumor tissue on the 3rd, 5th, and 7th days. The number of CFDA-SE^+^ DCs identified in tumor tissue and lymph nodes was dependent on the type of applied vaccine cells and the duration of the experiment. Our research showed that DC/IL-18 + IL-12/TAg infiltrated the tumor tissue the fastest and the most efficiently compared to other vaccine cells, while DC/TAg migrated the most quickly and effectively to the lymph nodes. The majority of the investigations on DC-based vaccines focus on improving DC activation; however, this research did not assess DC migration capacity. Migration capacity of vaccine DCs to tumor-draining lymph nodes or tumor tissue is one of the most important features conditioning the stimulation of T lymphocytes and thus the effectiveness of action [38]. This feature is influenced by the degree of maturity and activation of DCs as well as the site of vaccine administration.

Various routes of administration of DC vaccines have been tested: subcutaneous, intratumoral, intravenous, intradermal, intranodal, and recently, intralymphatic [39], and the clinically approved Sipuleucel-T vaccine is delivered intravenously [40]. Nevertheless, the most effective mode of DC delivery is still under discussion and may be related to the DCs' modification, the number of injected DCs, and essentially cancer type [41]. On the one hand, due to the phase of tumor microenvironment development, different types of immune cells can promote or inhibit cancer progression. Endogenous, TME infiltrating DCs can inhibit antitumor immunity by activating regulatory T cells in the tumor. In this case, recruitment and the presence of DCs in tumors are often correlated with poor prognosis [42]. On the other hand, DCs activated *ex vivo* with tumor-specific or associated antigens can cause a strong cytotoxic CD8^+^ T cell response against cancer. Therefore, to restore the capacity of the immune system for specific damage of the tumor, tumor antigen-activated DCs (in our work with a lysate of M38 cells) are increasingly used in therapy [43]. In our study, the effect of tumor antigen-activated DCs was enhanced by their additional genetic modifications for the production of IL-12 and IL-18. We observed that DC/IL-18 + IL-12/TAg was characterized by the best migration capacity to tumor tissue. Based on this observation, we can assume that these cells will most effectively recruit and activate T lymphocytes and NK cells to the tumor tissue and influence the cytokine balance in TME.

De Winde stated that when DCs have entered lymphatics, they require 24–72 h to reach the tumor-draining lymph nodes; however, the migration of DCs to tumor-draining lymph nodes is also controlled by the tumor microenvironment [41]. In the cancer-associated vasculature, expression of adhesion protein L1 is increased. This may favor the migration of immature DCs to the lymph node, which can lead to a tolerogenic response that supports the tumor's immune escape [44]. Additionally, the TME can inhibit DC migration to tumor-draining lymph nodes by overexpression of transforming growth factor-beta (TGF-*β*), which is associated with poor prognosis. TGF-*β* inhibits the expression of CCR7 on DCs, thereby leading to reduced ability of DCs to migrate to the tumor-draining lymph nodes [45]. CCR7 receptor present on the surface of DCs is one of the key factors affecting the ability of activated DCs to migrate [38]. Also, it has been found that a decreased number of DCs in tumor-associated lymph nodes may contribute to the formation of metastases [41]. In our research, we noted that DCs stimulated with tumor antigens, but not transduced, migrated most quickly to the lymph nodes.

Summarizing the obtained data, it would be worth considering the use of DC-based vaccines in therapy, consisting of DC/IL-18 + IL-12/TAg cells, which are characterized by an increased ability to infiltrate tumor tissue, and DC/TAg, which migrated most effectively to the lymph nodes. Thanks to this strategy, it would be possible to deliver vaccine cells to both tumor tissue and lymph nodes, thus delivering cells to the microenvironment, where they would support the activity of effector cells by cytokine production, and to nodes where they would activate virgin T cells by presenting antigens. Thus, the assessment of DCs' migration capacity is necessary to determine the antitumor efficacy of DC-based vaccines.

Multiparameter flow cytometry analysis of myeloid and lymphoid cells in tumors confirmed a high percentage of CD4^+^ and CD8^+^ T cells in the tumor tissue obtained from mice treated with DC/IL-18 + IL-12/TAg, 7 days after the administration of vaccines. Another important subpopulation which we investigated was macrophages. A statistically significant decrease in the percentage of TAM cells was observed in this study. This observation is extremely important in the context of the effectiveness of the applied therapy. It is believed that the high abundance of this cell population in cancer patients is associated with a poor prognosis. Moreover, this is associated with polarization towards M2 macrophages, which predominate among the TAMs in the tumor environment and secrete large amounts of IL-10 and TGF-*β*. These cytokines reduce the activity of NK cells, and the effector Th and CTL lymphocytes also inhibit the ability of DCs to secrete IL-12 [46].

Analysis of the systemic immune response showed that the administration of IL-18 and/or IL-12 transduced DCs additionally stimulated with tumor antigens induced an increase in the cytotoxic activity of CD4^+^, CD8^+^ T cells, and NK cells, which was associated with an increased percentage of cytolytic granule secreting cells. Moreover, we observed increased IFN-*γ* production in supernatants from restimulated splenocytes taken from mice receiving DCs producing IL-12 alone or in combination with IL-18.

The best confirmation of our postulate regarding the promising effects of dendritic cells transduced to the simultaneous production of IL-18 and IL-12 is the inhibition of the growth of MC38 tumors by slightly over 70% after double administration of cell vaccines. The use of such developed cell vaccines in a immunotherapy of MC38 tumor bearing mice involving triple administration of cell vaccines as well as in combination with chemotherapy may bring promising results. Research carried out by Tatsumi et al., who also transduced dendritic cells with IL-12 and IL-18 genes, but with the use of adenoviruses, confirm our observations showing the possibility of using such prepared cell vaccines in anticancer therapy [47].

The present work demonstrated that the single administration of DCs transduced for the production of IL-12 and/or IL-18 increases the influx and activity of CD4^+^ and CD8^+^ T lymphocytes and decreases CD11b cells into the tumor microenvironment. These phenomena proved to be dependent on the type of applied vaccine cells and the timing harvesting of tumors and tumor-draining lymph nodes. The transduction of dendritic cells to produce IL-12 and IL-18 simultaneously resulted in increased migration of these cells to the tumor nodule on the 3rd and 5th after the administration of cellular vaccines, whereas significant changes in the percentage of CD45^+^ cells infiltrating the tumor nodule were observed on day 7. Of note, each type of transduced cell vaccine was responsible for another piece of response activity; nevertheless, the highest effect was revealed when the vaccines consisted of DC/IL-18 + IL-12/TAg. Moreover, the anticancer response initiated by their single administration can be enhanced by repeated administration of these vaccines.

Thus, we postulate that the use of DCs able for simultaneous IL-12 and IL-18 production additionally stimulated with tumor antigens may generate a favorable impact on the activation of antitumor immune response, especially if these vaccine cells are administered repeatedly.

## Data Availability

The data used to support the findings of this study are available from the corresponding author upon request.

## Supplementary Materials

Supplementary Materials Supplementary Figure 1: Evaluation of NK and NKT cells in tumor tissue after a single administration of vaccine cells. (A) Percentage of NK cells in tumor tissue on the 3rd, 5th, and 7th days after single injection of DCs. (B) Percentage of NKT cells in tumor tissue. Supplementary Figure 2: Evaluation of NK and NKT cells in lymph nodes after a single administration of vaccine cells. (A) Percentage of NK cells in lymph nodes on the 3rd, 5th, and 7th days after single injection of DCs. (B) Percentage of NKT cells in lymph nodes. Supplementary Figure 3: Evaluation of CD4^+^ and CD8^+^ cells in the spleen after a single administration of vaccine cells. Supplementary Figure 4: Splenocyte phenotype after primary stimulation with genetically modified DCs unstimulated with tumor antigens. (A) Phenotypic characteristics of spleen cells obtained after 5-day coculture with genetically modified DCs unstimulated with tumor antigens. (B) The percentage of effector cells (CD107a^+^) was determined among CD4^+^, CD8^+^ T cells, and NK cells after 2-hour incubation with MC38 cells using flow cytometry.
