# Supplementary material for: The Beneficial Effect of IL-12 and IL-18 Transduced Dendritic Cells Stimulated with Tumor Antigens on Generation of an Antitumor Response in a Mouse Colon Carcinoma Model
Source: J Immunol Res. 2022 Mar 25;2022:7508928. doi: 10.1155/2022/7508928 (PMC8975686; doi:10.1155/2022/7508928)
Supplement: Supplementary Materials — Supplementary Figure 1: Evaluation of NK and NKT cells in tumor tissue after a single administration of vaccine cells. (A) Percentage of NK cells in tumor tissue on the 3rd, 5th, and 7th days after single injection of DCs. (B) Percentage of NKT cells in tumor tissue. Supplementary Figure 2: Evaluation of NK and NKT cells in lymph nodes after a single administration of vaccine cells. (A) Percentage of NK cells in lymph nodes on the 3rd, 5th, and 7th days after single injection of DCs. (B) Percentage of NKT cells in lymph nodes. Supplementary Figure 3: Evaluation of CD4+ and CD8+ cells in the spleen after a single administration of vaccine cells. Supplementary Figure 4: Splenocyte phenotype after primary stimulation with genetically modified DCs unstimulated with tumor antigens. (A) Phenotypic characteristics of spleen cells obtained after 5-day coculture with genetically modified DCs unstimulated with tumor antigens. (B) The percentage of effector cells (CD107a+) was determined among CD4+, CD8+ T cells, and NK cells after 2-hour incubation with MC38 cells using flow cytometry. [file 7508928.f1.docx]

Supplementary Material

## Supplementary Figures


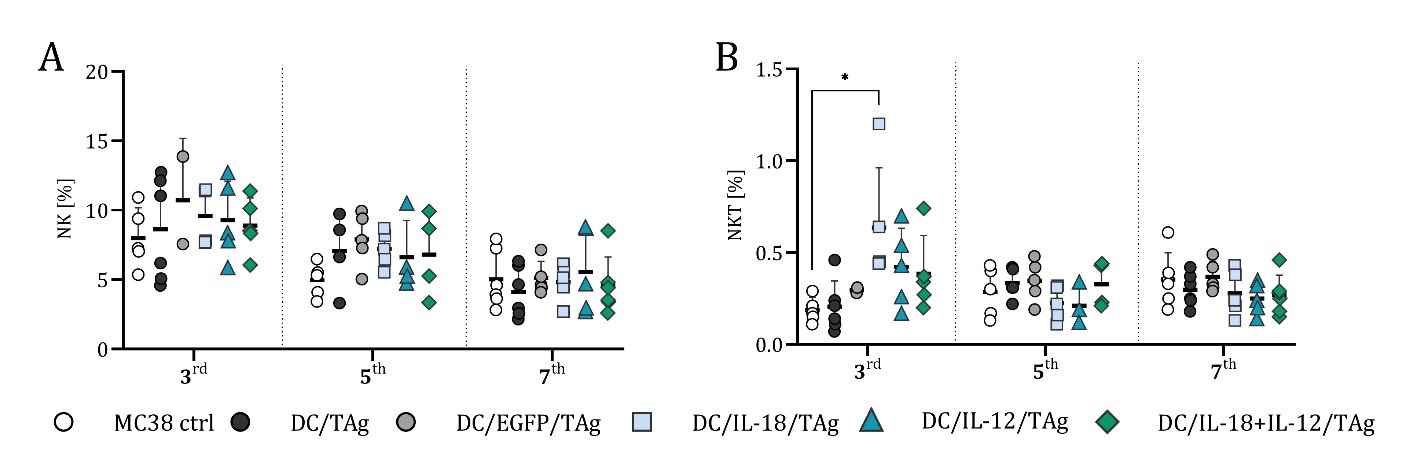


**Supplementary Figure 1.** Evaluation of NK and NKT cells in tumor tissue after a single administration of vaccine cells. (A) Percentage of NK cells in tumor tissue on the 3^rd^, 5^th^, and 7^th^ days after single injection of DCs. (B) Percentage of NKT cells in tumor tissue.


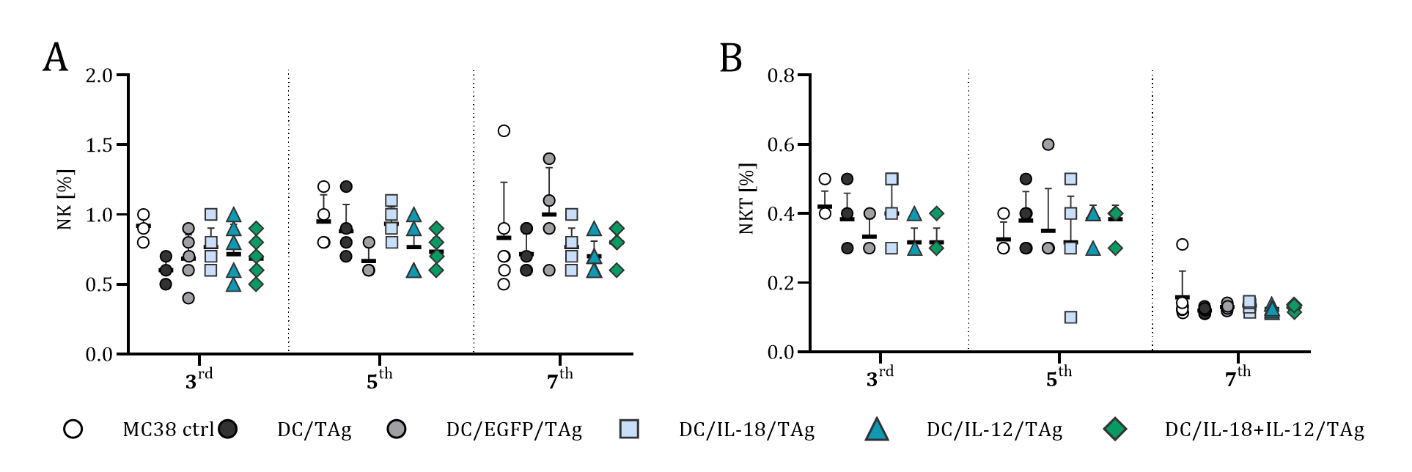


**Supplementary Figure 2.** Evaluation of NK and NKT cells in lymph nodes after a single administration of vaccine cells. (A) Percentage of NK cells in lymph nodes on the 3^rd^, 5^th^, and 7^th^ days after single injection of DCs. (B) Percentage of NKT cells in lymph nodes.


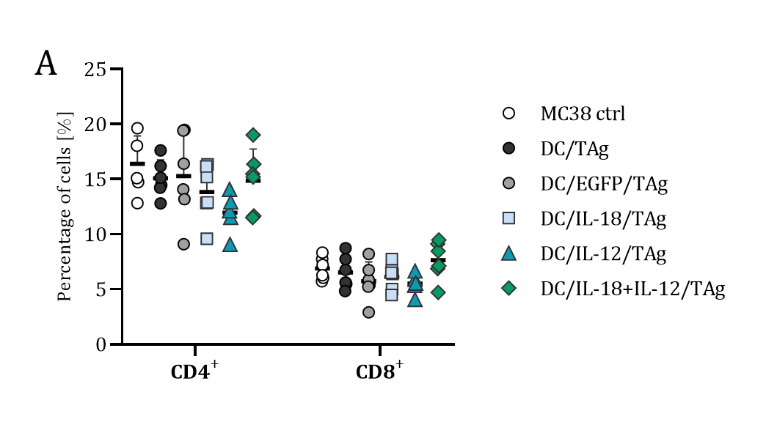


**Supplementary Figure 3.** Evaluation of CD4^+^ and CD8^+^ cells in spleen after a single administration of vaccine cells.


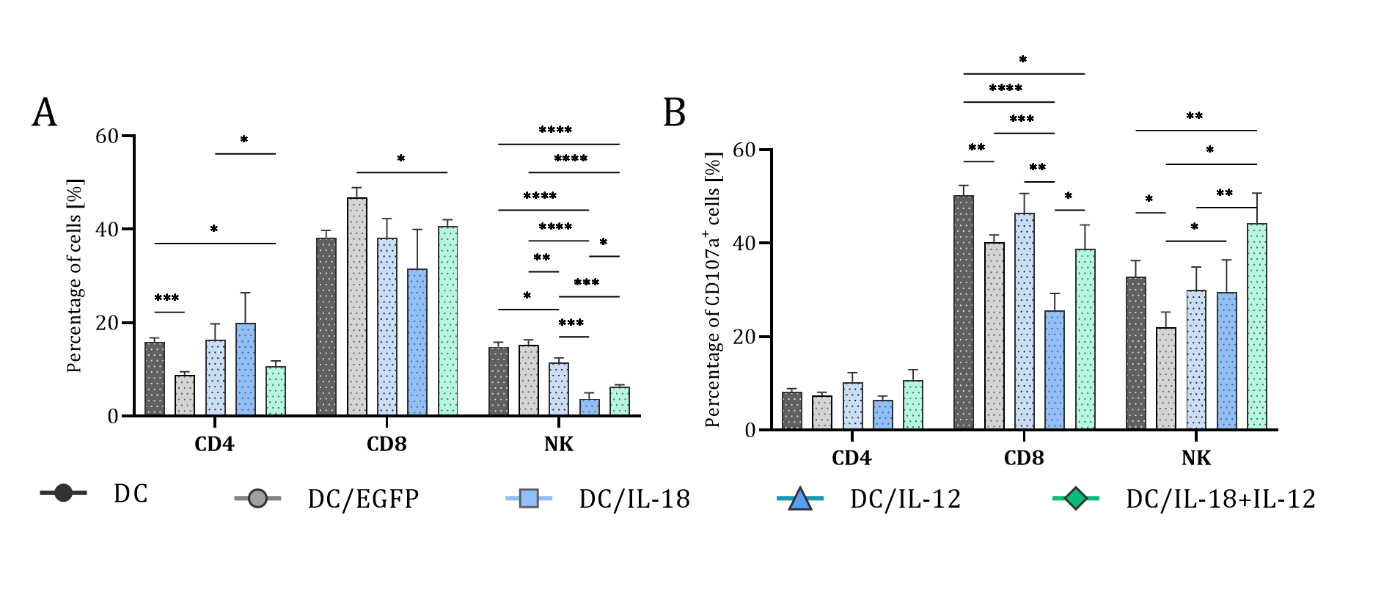


**Supplementary FIGURE 4.** Splenocyte phenotype after primary stimulation with genetically modified DCs unstimulated with tumor antigens. (A) Phenotypic characteristics of spleen cells obtained after 5-day co-culture with genetically modified DCs unstimulated with tumor antigens. (B) The percentage of effector cells (CD107a^+^) was determined among CD4^+^, CD8^+^ T cells and NK cells after 2-hour incubation with MC38 cells using flow cytometry.
